# Measuring capacity building in communities: a review of the literature

**DOI:** 10.1186/1471-2458-11-850

**Published:** 2011-11-09

**Authors:** Selma C Liberato, Julie Brimblecombe, Jan Ritchie, Megan Ferguson, John Coveney

**Affiliations:** 1 Menzies School of Health Research, Charles Darwin University, Darwin, Australia; 2 School of Public Health and Community Medicine, The University of New South Wales, Sydney, Australia; 3 School of Public Health, Griffith University, Brisbane, Australia; 4 School of Medicine, Flinders University, Adelaide, Australia

## Abstract

**Background:**

Although communities have long been exhorted to make efforts to enhance their own health, such approaches have often floundered and resulted in little or no health benefits when the capacity of the community has not been adequately strengthened. Thus being able to assess the capacity building process is paramount in facilitating action in communities for social and health improvement. The current review aims to i) identify all domains used in systematically documented frameworks developed by other authors to assess community capacity building; and ii) to identify the dimensions and attributes of each of the domains as ascribed by these authors and reassemble them into a comprehensive compilation.

**Methods:**

Relevant published articles were identified through systematic electronic searches of selected databases and the examination of the bibliographies of retrieved articles. Studies assessing capacity building or community development or community participation were selected and assessed for methodological quality, and quality in relation to the development and application of domains which were identified as constituents of community capacity building. Data extraction and analysis were undertaken using a realist synthesis approach.

**Results:**

Eighteen articles met the criteria for this review. The various domains to assess community capacity building were identified and reassembled into nine comprehensive domains: "learning opportunities and skills development", "resource mobilization", "partnership/linkages/networking", "leadership", "participatory decision-making", "assets-based approach", "sense of community", "communication", and "development pathway". Six sub-domains were also identified: "shared vision and clear goals", "community needs assessment", "process and outcome monitoring", "sustainability", "commitment to action" and "dissemination".

**Conclusions:**

The set of domains compiled in this review serve as a foundation for community-based work by those in the field seeking to support and nurture the development of competent communities. Further research is required to examine the robustness of capacity domains over time and to examine capacity development in association with health or other social outcomes.

## Background

Supporting the involvement of communities in efforts to enhance their own health has long been a strategy employed in the public health arena since it became recognised that traditional, top-down health directives have had minimal meaning for the communities concerned. Consequently these approaches has often resulted in little or no health benefits [1]. The enthusiasm to replace the old controlling approach with bottom-up community action has resulted in the development of a wide range of diverse projects and programs on the ground. The recognition of the merits of a community-based approach has become obvious in relevant policy documents, as for instance, those concerned with Aboriginal health in Australia [2,3].

The development of a set of attributes that enable a community to define, assess, and act on issues they consider to be of importance has been termed community capacity building although there are a variety of other related-terms with similar meaning [4,5]. For the purpose of this review, community is defined as "specific groups and networks of groups organising around specific issues, generally but not always spatially bound" [4].

Although the characteristics of a competent community were listed by Cottrell [6] more than 40 years ago, the primary drivers for acknowledging the importance of community involvement for health are enshrined in the World Health Organization's Alma-Ata Declaration on Primary Health Care of 1978, which states "The people have the right and duty to participate individually and collectively in the planning and implementation of their health care" [7]. This Declaration proved to be a watershed in including the concept of equity as a necessary concern of those dealing with the health of populations, and in formally giving voice to 'the people' when attempting to improve their health. The Declaration's underlying philosophy was reinforced in the next decade in the Ottawa Charter where the strengthening of community action was listed as an integral component of good health promotion action [8]. However, these two documents were high level calls to action and did not stipulate ways and means to incorporate bottom-up approaches in practice. Strategies for practical community capacity building were eventually highlighted as health promotion moved beyond lifestyle change to the creation of supportive environments for health [9]. The practical assessment of community capacity gained more impetus with the Healthy Community initiatives of the 1990s [10].

A range of advantages to the community has been specified as a result of community capacity building. The most recognised ones include: better reach of target population [11]; better use of resources [11,12]; increased local competence and commitment for health action and change [13] and increased community ability to respond to emerging health issues [5,10].

As these benefits are the result of the process of community capacity building, understanding its constituents or building blocks and being able to assess capacity development is paramount in facilitating capacity building in communities for social and health improvement. As indicated above, there are multiple understandings of community capacity with multiple domains having been identified to describe the characteristics of community capacity. For this reason, community capacity has proven difficult to measure [14] and its value often rendered invisible or under-estimated [15]. Consequently, identifying direct relationship between community capacity and positive health outcomes has remained limited.

To our knowledge no reviews have systematically examined and synthesised domains that are constituents of capacity building, especially in the community context. Given the increasing prominence of programmes integrating community capacity development into program delivery, a review of the literature in this area is required. Thus the current review aims i) to identify all domains used in systematically documented frameworks developed by other authors to assess community capacity building; and ii) to identify the dimensions and attributes of each of the domains ascribed by these authors and reassemble them into a comprehensive compilation. The underpinning purpose of this review is to identify appropriate domains to assess community capacity building in order to support successful project implementation in any situation, but particularly those addressing health in an Australian Indigenous remote community context. Community capacity for the purpose of this review is taken to mean those local initiatives that may or may not be embedded in community organisations and that concentrate on specific health or social concerns [16].

## Methods

Given the complexity of community capacity building and the often convoluted processes used within each program to assess this capacity, this review was strongly influenced by Pawson's overarching protocol of realist synthesis [17]. This approach suggests that the search process should be as tight and systematic as in conventional systematic reviews but Pawson makes clear the primary purpose of the effort is to contribute to the building of explanatory theory through a process of synthesis. Whereas conventional systematic reviews try to answer the question 'Does it work?', a realist synthesis approach attempts to investigate 'Why does it work?' and 'How does it work?' In this review, there were a few differences from Pawson's approach including being less iterative in the review steps and having more specific inclusion and exclusion criteria, especially choosing only to include studies published in peer-reviewed journals. The aim of this review was to synthesise what others have found in the practice of assessing community capacity, particularly investigating what are the various domains deemed important and, how they have been developed and applied. The end result is a set of synthesised domains as a useful conceptual tool rather than the full formulation of an actual theory.

### Search strategy

Electronic databases including Web of Science, PubMed, Science Direct and EBSCO were searched on 24^th ^June 2010. Search terms included "Community based participatory research", "Community participation", "Capacity building" and "Community capacity" in the title field combined with "Evaluat*" or "Measure*" in the abstract field.

### Inclusion and exclusion criteria

The articles included studies of the assessment of capacity building or community development or community participation.

Types of study design considered in the review included: i) any type of study assessing community capacity building using framework based on a-priori models where the domains were not modified or developed for a specific context; and ii) any type of study based on emergent models developed for implementation and evaluation within a specific context.

Papers were included if they met at least two of the three following criteria:

- the capacity building domain measures were theoretically and/or empirically informed;

- the measures showed evidence of community involvement in their development;

- the measures were applied in the field.

Additionally, papers had to meet a minimum of four of seven criteria [18] that included suitability of the methodology to the research question, a description of sampling selection, a description of data collection and analysis, evidence of applying rigor (coding by two or more coders), triangulation, reflexivity and relevance (researcher and research process).

Papers were excluded if they meet any of the following criteria:

- any existing reviews;

- any studies measuring organisational capacity rather than community capacity

- any studies assessing research capacity;

- studies not published in English; and

- studies not published in peer-review articles.

The initial search strategy yielded a total of 1114 papers. Screening occurred at two levels, with the first based on title and abstract as judged by three authors (JC, JR and SL). A cross check was performed with 30 papers screened by these three authors who identified the same relevant papers. Each author screened one third of the total 1114, reducing the number to 94 articles. A further 17 references were added, identified from the reference lists and from professional contacts. Copies of 108 papers were obtained. Eight papers were excluded as they were either not published in English (4) or not in peer reviewed journals (4). Screening at the second level was based on the full paper, with those included mentioning capacity building measuring terms/domains. A total of 54 papers which explored the constructs of community capacity or applied a framework for collective community action were included. Nine reviews [4,15,19-25], nine studies that measured organisational capacity rather than community capacity [9,12,26-32] and one study assessing research capacity [33] were excluded. This left 35 papers for further review.

### Data extraction

A template was developed by the authors to perform data extraction, seeking information on:

- aims of the study;

- domains or terms assessing community participation or community capacity;

- level of community participation in the development of the framework;

- context; and

- practical application in the field.

Papers were classified into two groups:

- Group 1 based on existing models and applied to a project for assessing community capacity building

- Group 2: based on models arising from the implementation and evaluation of a particular project.

## Results

Of the 35 papers selected, 17 [34-50] did not meet the criteria. Only the latest [51] out of two studies [51,52] from the same author was included and therefore 17 papers were reviewed comprehensively with the domains used to assess capacity building identified, and summarised, before being reassembled through synthesis.

### Group 1

Ten studies were included. Each of them was based on one of seven existing models where the domains used to assess community capacity building were not modified or developed for a specific context. Details of these studies including the domains identified by the authors are given in Table 1 (Additional file 1). The following provides a very brief description of the seven models and an indication of the main contribution from these papers.

#### Hawe model

The Hawe model was first described in 1990 and drew from community development and practice based research, literature on learning organisations, and the experiences of earlier cardiovascular disease prevention initiatives. Hawe et al have highlighted the importance of context in the assessment and development of community capacity and viewed capacity building as representing a multiplier effect rendering a community more competent to not only address the problem of interest but able to tackle other issues. The two Australian studies that drew on the Hawe model were by O'Meara et al [53] evaluating a project aiming to "revitalise" a small rural community, and Yeatman et al [54], reporting on the results of an assessment of the capacity of an organisation to support core skills in health promotion.

#### Rifkin model

The Rifkin model was developed in 1988 to depict the level and quality of community participation schematically as a spidergram. Drawing on 100 case studies, Rifkin ascertained that the community participation process was influenced by five domains: identifying the need of the communities, representation of interest groups in the organisation of the program, form of leadership, mobilization of resources, and structure of program management. The model was designed to enable analysis of change enabling capacity development to be quantified and thereby linked with outcomes [55]. Three papers [13,55,56] using the Rifkin framework were identified. Ui et al [56] aimed to identify factors facilitating community participation in health centre management. Chilaka [55] described an innovative method to quantify capacity development and relate to health outcomes while Andersson et al [13] aimed to understand the development of inter-sectoral participation in three municipalities implementing diabetes prevention programme interventions.

#### Goodman/Labonte/Laverack/Fawcett model

The framework of Goodman/Labonte/Laverack/Fawcett was first described in 2000. These authors have explicitly drawn from each others' work and/or collaborated in developing capacity building domains. The studies captured by this review drawing on this pooled model are mostly commentaries that describe the characteristics of capacity domains and/or provide interesting insight into the application of domain measures. The only study [57] meeting the inclusion criteria aimed to identify the dimensions of community capacity that were enhanced as part of a community-based participatory research program.

#### Foster & Fishman model

First described in 2001, this model was used in only one study [58] identified in this review. This evaluation study described the steps taken to develop and evaluate the activities of an international network promoting collaborative capacity among regional partners involved in activities related to the prevention of labour discrimination towards immigrants. Survey, interview and discussion forum methods were used.

#### Moore model

The Moore et al model was first described in 2006 and aimed to deliver biodiversity conservation outcomes. Model development was based on a literature review and synthesis, with subsequent refinement using interviews [59]. The cognitive and structural dimensions of social capital and knowledge, skills and experience dimensions of human capital were identified as important elements of community capacity. This model was used in two studies identified in this review [51,52], both by Robins. The latter study [51] was chosen to represent this model as the measures used were extended from the former study. Robins aimed to give a practical meaning to capacity building through identifying measures, placing these measures within a broader systems framework, and exploring stakeholder feedback on specific measures to inform framework implementation. The focus was on natural resource management and drew from both the health sector and the risk and emergency management sector primarily in Australia in developing the domains. Twenty two measures were originally presented in a discussion paper to stakeholders and an additional seven measures identified by workshop participants and survey respondents.

#### Johnson/Sofaer model

This model aimed to support people working in community health coalitions by providing insight about the nature and development of these coalitions. The model was based on a synthesis of the characteristics of effective groups developed by Johnson and Johnson [60] adapted from Sofaer [61]. The model included organizational structure, resources, leadership and decision-making procedures as factors which could help make coalitions more productive and lead to constructive conflict resolution. Trust, adaptation, and dedicated staff were found to contribute to sustainability. One article [62] was based on this model. Through a multi-site case study Schulz et al [62] evaluated group dynamics in three community-based participatory research interventions that aimed to increase the responsiveness of local health departments to communities and to improve family and community health addressing social determinants of health. The application of the evaluation tool on an ongoing basis provided a structured opportunity for members of the coalitions to reflect on group interactions, and to engage in collective problem-solving regarding group effectiveness.

#### Active Partners Benchmarkers model

The Active Partners Benchmarkers model [63], first described in 2003, includes twelve benchmarks for communities and public policy makers to assess the extent to which community participation is taking place. The twelve benchmarks are listed in relation to the four key dimensions of participation including influence, inclusivity, communication and capacity. This model was used to assess capacity building in one study [64] which aimed to develop a self-assessment tool for organizations to evaluate the quality of community involvement.

### Group 2

Seven studies, detailed in Table 2 (Additional file 1) identified domains of community capacity building which were developed while implementing and evaluating particular projects. The domains in these studies were developed drawing from either the literature [5,65-67] or case studies [10,16,68]. Four of these studies [10,66-68] tested the developed domains in the field.

Jackson's study [65] highlighted the importance of considering the socio-environmental conditions that may impede or facilitate capacity development. According to Jackson "people in communities have many talents and skills and accomplish many things together" and community capacity relates to action accruing from collective action rather than being an aggregate of individual abilities. Key factors supporting community action include: a positive social environment (caring neighbours, strong sense of community, celebratory events); and the ability to work together, link to one another and participate. Factors hindering collective work included: a negative public image of the community; high levels of individual stress in trying to meet basic needs; and policies and regulations set by governments. Supporting and hindering factors were diversity, physical environment, community infrastructure and agency characteristics. From Jackson's viewpoint, indicators have to be measurable, positive and yet responsive to individual community uniqueness. Jackson developed a framework to assess community capacity through workshops, focus groups and interviews with residents and agency workers.

Lempa et al.'s study [16] aimed to assess local public health initiatives and found that capacity was hard to grasp from one vantage point as there were differences in those capacity-building elements considered important by leaders when compared to those identified by participants. The domains to assess community capacity were generated through pilot-testing and cross-analysis of multiple case studies, producing a survey which was then applied to different sets of stakeholders.

Lennie's study [66] aimed to achieve long-term sustainability of the Healthy Community Initiatives project through increasing partnerships and linkages, enhancing capacity in participatory monitoring and evaluation, increasing participation and ownership in activities, supporting empowering forms of leadership and developing learning communities. The methods to develop the framework to assess community building included a review of the literature, holding meetings with experts and conducting focus groups to provide feedback on the framework.

Littlejohns et al [10] identified key elements of community capacity specifically related to a rural heart health project. The development of the framework involved meetings with working group members to gather information regarding working effectively in their community and pilot-testing the framework with 120 community members.

Maclellan-Wright et al [5] developed a framework based on a literature review of existing measures and a national meeting of experts to further inform community capacity domains. Two focus groups were used to test face and construct validity and finally pilot testing of the instrument was carried out with 114 community organizations.

Yassi et al [67] assessed a community participatory process in a multisectoral intervention. The development of the framework included focus groups to discuss evaluation strategy, interviews with leaders of community organizations to determine the level of participation of leaders and the community, and a survey focused on individual perceptions of the interventions. This information was used to design the instrument which was then used to gather information on the views of community members regarding the level of community participation. Community members were actively involved in the gathering of the information and were trained to conduct interviews.

Zacocks and Guckenburg' study [68] aimed to identify factors that foster community capacity to combat social problems. The capacity of 13 coalitions was examined. The development of the framework to assess community building was based on changes in programs, services or policies among eight types of organisations and included a literature review, analysis of program and evaluation documents, semi-structured interviews with key staff members and a survey with the decision-making body.

### Reassembled domains derived from the 17 studies assessing capacity building

Multiple domains identified in both Groups 1 and 2 were used to assess capacity building. Many overlaps and commonalities among these domains were found and, through a process of considering the way each author described the characteristics of each domain, they were reassembled into nine comprehensive domains with six sub-domains. Table 3 (Additional file 1) provides definitions for each of these domains, drawing on the domain features and characteristics described by the authors.

"Learning opportunities and skills development" featured strongly in most of the studies, as would be expected in attempts to build the capabilities of communities and strengthen teams. "Resource mobilization" was considered very important by most, always with the term including the attainment of funds but also referring to drawing on people, structures and systems. "Partnership/linkages/networking" was seen to be essential both in the terms of more equal relationships but also in "linkages and networking" within and across communities. "Leadership" was described as essential in motivating communities to participate towards a goal, in negotiating conflict and in overcoming obstacles. "Participatory decision-making" tended often to be seen as complementary to leadership in working with a range of community viewpoints regarding identifying issues of concern and ways to address these issues. All of these domains were represented in more than half the models or frameworks of the reviewed papers.

Less frequent, but still identified often enough to be included, were the remaining four with some having sub-domains. An "assets-based approach" emphasised the importance of starting with community strengths already present. A "sense of community", where positive perceptions of the community itself were apparent, was identified by many authors, with its sub-domain being "commitment to action" where communities felt responsible to act for their own good. "Communication" was valued, with a sub-domain of "dissemination" often identified. Finally, many authors referred to the importance of a "development pathway" for building capacity, with this domain having four sub-domains - the presence of "shared vision and clear goals", the inclusion of a "community needs assessment" and the "processes and outcome monitoring" and a recognition of the importance of positive "sustainability" as opposed to negative project termination.

The way in which the original domains identified in the studies included in this review were regrouped into nine domains and six sub-domains is shown in Tables 4 (Group 1) and 5 (Group 2) (Additional file 1). Many domains were named differently by different authors. For example, "learning opportunities and skill development", was referred to as "group empowerment" [62] and "growing the knowledge base" [51].

Some domains identified by some authors were found to represent more than one domain in this review and therefore were reassembled into two domains. For instance "commitment and resource mobilization" identified by Ui [56] was reassembled into: "resource mobilization" and "commitment to action". "Working together towards a common purpose" identified by Jackson [65] was also found to represent two domains: "partnership/linkages/networking" and "shared vision and clear goals".

On the other hand, some domains identified by some authors were found to overlap and were reassembled into one domain. For example, "democratic leadership" and "leader with adequate conflict-resolution skills" identified by Garcia-Ramirez [58] were both reassembled into "leadership".

## Discussion

Seventeen articles were included in this review and from these articles nine domains were identified to be used in the assessment of community capacity building: "learning opportunities and skills development", "resource mobilization", "partnership/linkages/networking", "leadership", "participatory decision-making", "assets-based approach", "sense of community", "communication", and "development pathway". Six of these domains were commonly used to assess community capacity in various contexts, however there was less consensus concerning the domains "sense of community", "assets-based approach", and "communication". Six sub-domains were also identified: "shared vision and clear goals", "community needs assessment", "process and outcome monitoring", "sustainability", "commitment to action" and "dissemination".

Domains used in the contemporary context appear to be very similar to that defined by Cottrell [6] in the 1960s to represent a "competent community": i) community participation in defining and reaching goals; ii) commitment; iii) community understanding of its own and other's issues; iv) articulateness of the community in expressing its needs; v) effectiveness in communicating information and achieving consensus within a community; vi) conflict management; vii) management of relations within the community including the use of outside resources; and viii) representative decision-making. These domains have also been described by Israel as the nine factors that influence community empowerment [20]. The similarity of the domains identified by this review to those defined by Cottrell [6] 40 years ago suggests that concepts of capacity development have little changed over time.

It is evident from this review that a set of core domains for assessing capacity building are relevant across different contexts with differences depending on the context and the purpose. A further difference is in how domains are assessed. For example, in some cases domains are assessed broadly allowing new perspectives to emerge through the process of capacity building, whereas in other cases specific components of domains are assessed.

The core domains frequently used to assess community capacity: "resource mobilization", "partnership/linkages/networking/", "participatory decision-making" and "leadership" were included in Cottrell's original eight dimensions. Studies that applied these pre-existing domains, and tested relevance [28], and construct validity [28] agreed with the value and appropriateness of these domains.

It is noteworthy that the one study that used qualitative methods to elicit dimensions of community capacity from the perspective of community members within a marginalised community reported the less frequently used domains as important indicators of community capacity. Community members were asked to describe their community, the talents and strengths of their community, and the enablers and challenges to working together. Factors that related to "sense of community" and "assets-based approach" were emphasised [65]. Littlejohns et al who also worked closely with members of marginalised communities included 'sense of community' as an important indicator of community capacity [10]. These domains may be more relevant in the context of marginalised communities.

Aspects of "communication" were considered within domains such as "participatory decision-making" (ie. reaching a consensus, problem-solving as a team) and "leadership". However using the Rifkin model, Anderson considered "communication" as important as the other domains [13]. Littlejohns also included communication as a stand-alone domain [10].

This review found that different terms for domains with similar meanings were used by authors depending on the context and the purpose of the project within which community capacity was measured. For example, Garcia-Ramirez et al in assessing the capacity development of collaborations, assessed elements of the "partnership/linkages/networking" domain that related to the capacity of teams to work together effectively and the relationships that the collaborations formed with communities. These domains were referred to as "team work" and "relationships with communities" [58]. On the other hand, Lempa et al who examined the relevance of domains from the perspective of leaders and non-leaders, included items that related to "external networking" and "networking internal to the community" within the "partnership/linkages/networking" domain [16].

A further difference is in how domains are assessed. The depth and extent by which domain dimensions are assessed depends on the purpose of the project and the characteristics of the target population. Within the context of community development that authors such as Laverack, Gibbon and Jackson have described, there is merit in supporting community members to define the characteristics of each of the domains.

Lastly there are differences in the interpretation of some domains by different authors. "Sense of community", "community history" and "community values" were identified by Goodman et al as central to community capacity development, with "community power" included as a distinct domain [40]. Goodman's use of "community power" however differed from its use in the Rifkin model where each domain is assessed against a continuum of increasing community empowerment in association with capacity development.

When a-priori models have been used, authors have noted limitations. For example, "equity in participation", including women's involvement, was considered an important aspect of "community participation" that was not incorporated in the Rifkin model [38]. Lennie [66] cautioned against the potentially unintended disempowering consequences of capacity building if equitable opportunity for learning and participation is not considered. Using the Hawe model, Yeatman and Nove [54] reported "commitment to action" important in addition to Hawe's five domains (organisational development, workforce development, resource allocation, partnerships and leadership). Lempa [16] also demonstrated the importance of including different stakeholders in the assessment of community capacity as different groups of people have different perceptions of community participation. Futhermore, the socio-political context of the community capacity needs to be considered as capacity development is both enhanced and constrained by socio-political conditions [55].

A range of advantages to the community have been mentioned as a result of community capacity building such as better reach into the target population [11], better use of resources [11], increased community ability to respond to emerging health issues [5], increased local competence and commitment for health action and change [13] and stronger community action to impact on priority determinants of health [10].

In reviewing these 17 studies, the focus was found to be much more on the development of relevant domains and a description of the process of community capacity building rather than on the specific measurement of the process. For example, there are few examples of capacity building being measured longitudinally. This lack of evidence has resulted in lack of guidance on the relative importance of domains, the feasibility and benefits of long-term assessment of capacity building, the relationship between domains over time and to what extent measures of capacity development can be associated with health outcomes [57].

Maclellan-Wright, Rifkin and Laverack have made important contributions to the development of processes to actively engage the community in measuring its capacity. These authors suggested that capacity measures be developed in the context of specific programs, be culturally specific, not burdensome, and be applied in a group setting, used at various stages of projects - project planning, implementation and evaluation, and used as a guide to identify project strengths and weaknesses. Such indicators have to be measurable, positive and yet responsive to individual community uniqueness [65].

Authors who have elsewhere contributed substantially to the development in understanding of community capacity have drawn from experience and practice largely within the area of health-related concerns. A strength of this review is that it has endeavoured to widen its net to review literature beyond that of health promotion. our review may have been limited in that it only examined literature available in English leading to us possibly overlooking a number of papers or reviews that are available in other languages. Similarly, our review was limited to peer-reviewed publications and did not take account of the 'grey' literature. Again, this may mean we have overlooked some important studies in this area. However, notwithstanding these limitations, this review provides a first time account of the assessment of community capacity building. We believe that the review will be of use to researchers and practitioners who, like us, are attempting to build community capacity and to assess the on-going development of community skills in this area. Our own work focuses on the development of community capacity building measurements in Australian Indigenous communities. However, there are clear applications to other groups who are part of capacity building enterprises and projects.

## Conclusions

Like already highlighted by Labonte, there is consensus on a set of key domains and less consensus on fewer of the domains. The use of domains depends on the context and purpose of the capacity building process. All domains used in various contexts in the health and non-health sectors to assess capacity development have been considered and common characteristics described. A set of nine domains used to date in different contexts were identified providing a guide to consider domains in assessing capacity building in communities. These are broad domain areas that provide a definition of the key elements. However sub components of these domains can be constructed to guide capacity development in particular areas to achieve specific outcomes.

The outcomes of this review are twofold. First and specifically, the set of domains and sub-domains identified are to be used to assess community capacity building to support healthy eating in an Australian Indigenous remote community context as part of the Good Food Systems, Good Food for All Project. All review authors are involved in this Project which aims to trial a monitoring and evaluation learning approach to assist community based organizations and services in remote Australia. The project aims to improve the food system and services these agencies deliver to provide an affordable and healthy food supply, with community capacity building being central to the approach. Second and more generally, it is hoped that this systematic review will serve as a foundation for community-based work by others in the field in seeking to support and nurture the development of competent communities.

Further research is required to examine the robustness of capacity measures over time and to examine capacity development in association with health or other social outcomes. For policy-makers and practitioners to embed the measurement of capacity development within programs and initiatives, it is imperative that high quality research continue to be conducted in this area.

## Competing interests

The authors declare that they have no competing interests.

## Authors' contributions

SL undertook the literature search, data extraction and data analysis; helped with data screening, data collation, quality assessment of the studies and drafted the manuscript. JB secured support for this review, helped with quality assessment of the studies, data collation, data analysis and manuscript writing. JR assisted in the design of the study, helped with the data screening, data collation and writing of the manuscript. JC defined the design of the review and helped with data screening and data collation. MF helped with data collation. All authors read and approved the manuscript.

## Pre-publication history

The pre-publication history for this paper can be accessed here:

http://www.biomedcentral.com/1471-2458/11/850/prepub

## Supplementary Material

Additional file 1 **Table 1 - Studies based on seven existing models (Group 1) assessing community capacity building**. Table 2 - Studies describing original frameworks (Group 2) to assess community capacity building. Table 3 - Definitions of the reassembled domains and sub-domains as synthesised in 17 community capacity building studies. Table 4 - Domains used by Group 1 authors and their correspondence with reassembled domains assessing community capacity building. Table 5 - Domains used by Group 2 authors and their correspondence with the reassembled domains assessing community capacity building. Click here for file
