# Supplementary material for: Measuring capacity building in communities: a review of the literature
Source: BMC Public Health. 2011 Nov 9;11:850. doi: 10.1186/1471-2458-11-850 (PMC3229539; doi:10.1186/1471-2458-11-850)
Supplement: Additional file 1 — Table 1 - Studies based on seven existing models (Group 1) assessing community capacity building. Table 2 - Studies describing original frameworks (Group 2) to assess community capacity building. Table 3 - Definitions of the reassembled domains and sub-domains as synthesised in 17 community capacity building studies. Table 4 - Domains used by Group 1 authors and their correspondence with reassembled domains assessing community capacity building. Table 5 - Domains used by Group 2 authors and their correspondence with the reassembled domains assessing community capacity building. [file 1471-2458-11-850-S1.DOC]

# Tables

**Table 1 - Studies based on seven existing models (Group 1) assessing community capacity building**

| **Reference** | **Domains identified to assess community capacity building** | **Aim of the study** | **Study design and framework development** | **Community involvement** | **Practical application in the field** |
| --- | --- | --- | --- | --- | --- |
| O’Meara et al (2004)[1] | - Vision and leadership - Structure and partnerships - Community engagement - Resources | To evaluate a 3-year community capacity building project | 3-year longitudinal study based on Hawe (1997) with domains modified later | Not mentioned | A 3-year community capacity building project |
| Yeatman & Nove (2002) [2] | - Leadership - Partnership - Building, demonstrating and maintaining commitment | To present a case study of the application of a framework for capacity building | Case study – long term follow-up based on Hawe (1997) | Not mentioned | Case study included interviews, document analysis and focus groups. |
| Andersson et al (2005) [3] | - Planning of activities, identification and correspondence of needs - Resources: mobilization and contributors - Leadership: interest groups in the steering committee - Network: partnerships, mobilisation of partners and stage of development - Implementation: responsibility, the steering committee’s role | To understand the development of inter-sectoral participation in three intervention municipalities using the Rifkin spidergram method | Case studies over 5 years based on Rifkin (1988) | Country council, municipal head, municipal administrations, local health units, primary health care, social welfare authorities, nongovernmental organizations and food store | Steering committee formation is described, local participation assessed by open-ended questionnaires, changes over time are presented in group discussions and cross- comparison analysis |
| Chilaka (2005) [4] | - Needs assessment - Community organization - Resource mobilization - Programme management - Political commitment | To outline the process involved in the estimation of quantitative values to measure community participation, and to compare levels of community participation in 5 African countries | Structured review. Participation was assessed based on Rifkin (1988) | Not mentioned | The scores and rates for community participation in 5 African countries were derived from assessment parameters based on country profiles and reports of programme activities |
| Ui et al (2010) [5] | - Community representation - Commitment and resource mobilization - Decision making: expressing opinions, problem solving power and needs assessment - Management, supervision, work information shared - Community accountability, recognition of roles and information sharing | To identify factors facilitating community participation in health centre management | Questionnaire survey based on Rifkin (1988), field survey, interview with stakeholders | Not mentioned | 8 case studies in nongovernmental organizations were evaluated |
| Parker et al (2010) [6] | - Leadership - Participation - Skills - Resources - Social and organizational networks - Sense of community - Understanding of community history - Community power - Community reflection | To identify the dimensions of community capacity enhanced as part of a community-based participatory research community health development approach | Literature review and one-on-one semi-structured, in-depth  interviews to modify dimensions to assess capacity building based on Goodman et al. (1998) | Organization representatives, university-affiliated research team members, key  community organization staff and community  members | Project evaluated using a participatory and formative evaluation approach in which members were active participants in design and implementation of evaluation activities including instrument development and interpretation of the results |
| Garcia-Ramirez et al (2009) [7] | - Teamwork skills - Understanding due to participation - Productive team work - Members’ viewpoints respected and valued - Capability of influencing workgroup - Participation of all members is encouraged - Identification and strengthen of skills - Learning to overcome difficulties - Positive and open atmosphere - Effective conflict-management atmosphere - Shared goals - Participation in decision-making process - Interests and ideas from all were considered - Individual differences were taken into account - Relationships with communities - Social networking - Democratic leadership - Leader with adequate conflict-resolution skills - Work procedures - Communication - Shared information and solved problems by consensus - Optimized use of resources - Continuous improvement and feedback - Deadlines respected | To describe the steps taken to develop and evaluate the activities of an international network promoting collaborative capacity among regional partners | Collaborative relationship among partners was established, collaborative capacity was built, and was evaluated, based on Foster and Fishman (2001) | Not mentioned | Collaborative capacity of the network was evaluated. |
| Robins (2008) [8] | - Define the organizational business - Set the standards & expectations - Access infrastructure & resources - Strengthen structures & processes - Underpin with effective business systems - Develop and sustain networks & relationships - Growing the knowledge base - Build the information base & tools | To give meaning to capacity building through identifying practical measures, placing these measures within a broader systems framework and exploring stakeholder feedback to inform framework implementation | Literature review, discussion paper, feedback from presentations, consultations and a workshop and a survey, based on Moore (2006) | Representatives from regional, policy/research and Indigenous interest groups | Not mentioned |
| Schulz et al (2003) [9] | - Leadership and participation - Comfort level for expressing opinions - Addressing conflict problems - Decision-making procedures - Working together as a group to solve problems - Level of influence and power of self and others in the group - Perceived level of trust; - Meeting organization, agenda setting, facilitation, and staffing - Accomplishments / impact of group; - General satisfaction - Personal, organizational, and community benefits of participation; - Member background and meeting attendance - Sense of ownership / belonging to the group: cohesion - Group empowerment - Community empowerment | To identify reasons for the evaluation of partnership dynamics, and the selection of relevant dimensions for evaluation and assessment. | Domain terms adapted from Sofaer/Johnson (1982) | Not mentioned | Application of evaluation instrument in three case studies |
| South et al (2005) [10] | - Community diversity - Organizational procedures - Communication and flow of information - Staff support to engage with communities - Opportunities in decision making process - Resources access and control by communities | To develop a self-assessment tool for evaluating community involvement | A longitudinal study. A multi-agency working group developed a tool. Evaluation resources were reviewed through a literature review based on the Active Partners Benchmark Model (2000). Experts were consulted and the tool was piloted | Feedback from piloting the tool was incorporated in the tool | Tool was piloted in two primary care trusts. |

**Table 2 – Studies describing original frameworks (Group 2) to assess community capacity building**

| **Reference** | **Main elements related to community capacity / participation** | **Aim of the study** | **Study design and framework development** | **Community involvement** | **Practical application in the field** |
| --- | --- | --- | --- | --- | --- |
| Jackson et al. (2003) [11] | - Community is supportive of diversity - Positive perceptions of their communities - Residents celebrate together - Active participation in the social, political and economic life of the community - Working together towards a common purpose - Participation from all parts of the community - Sense of control and sense of ownership of programs | To develop and evaluate a model of community capacity | Literature review, formation of a coalition and community advisory committees, interviews, focus group and development of indicators of community capacity | Coalition formed by workers and residents. Community advisory committees included residents and community agency workers | Not mentioned |
| Lempa et al. (2008) [12] | - Leadership - Resources - External networking - Visibility/recognition - Ability and commitment to organizing action - Personnel sustainability - Communication with community members - Relationship with influential others | To describe the development of two capacity measures of local public health initiatives for leaders and non-leaders. | Multiple case studies, group interviews, cross-case analysis, survey, meeting and pilot-testing of measures in a local public health initiative | Local leaders representing community initiatives | Not mentioned |
| Lennie (2005) [13] | - Social empowerment (new knowledge and information, awareness and understanding of issue, skills, abilities and competence, support, friendship and inspiration, participating in group activities with women and networking) - Political empowerment (having a voice and being listened to, participating in policy-making, taking action to change your life or your community, networking and lobbying and changing stereotypes about rural women) - Technological empowerment (knowledge, awareness, understanding, skills, competence and ongoing support and advice in using communication and information technology, access to high-quality technologies, confidence to use and speak about it). - Psychological empowerment (self-confidence and self-esteem, feeling more valued and respected, motivation, interest and enthusiasm, freedom to do things or express yourself, feelings of belonging and well-being and happiness) | To present results from the implementation of a process that aimed to build the capacities of people to evaluate their local communication and information technology initiatives | Literature review, meetings with expert researchers and collaborating partners, and focus groups with local people | Researchers, collaborating partners and local people | Two case studies presented. |
| Littlejohns et al. (2000) [14] | - Shared vision - Quality of human relationships that make it possible for people to live together in a healthy and sustainable way - Resources - Knowledge and skills - Participation - Leadership - Communication - Critical learning | To evaluate a community capacity assessment process | Review of evaluation methods for health promotion projects in the region, identification of elements or domains of community  capacity, redefinition of the capacity domains | Core group of a health community initiative and local residents | Core group planned and hosted a half-day community meeting with 120 community members |
| Maclellan-Wright (2007) [15] | - Participation - Leadership - Community structures - Role of external supports - Asking why - Resource mobilization - Skills, knowledge and learning - Links with others - Sense of community | To explore how community capacity has been defined and measured and to identify validated community capacity measurement instruments | Literature review and national think tank with 21 experts to inform capacity community domains, focus groups to inform content validity and pilot testing | Community practitioners informed content validity and domains pilot tested with community organizations | Not mentioned |
| Yassi (2003) [16] | - Opportunity to participate - Linkage systems - Information and communication patterns - Joint decision making - Mobilization of people and resources - Self-definition of interests by the local community | To describe an evaluation of a community participatory process | Information form a literature review, interviews, community workshop and household surveys were used to design the evaluation | Key informants, community members | A multisectoral intervention to address a  variety of health determinants is described |
| Zacocks (2007) [17] | - Resource (funding) - Lead agency (supportive, power broker) - Governance (stability, participation from organizational representatives, participatory decision making) - Leadership (collaborative style, project director effectiveness, stability) | To identify coalition factors (resources, lead agency, governance, and leadership) that foster organizational capacity | Retrospective multiple-site case study using informant interviews, program and evaluation documents, and surveys of steering committee leaders, members, and project directors. Narrative summaries were drafted, coded into categories and a scale created to assess capacity | Not mentioned | Organizational capacity and coalition factors were identified in 13 sites |

## Table 3 – Definitions of the reassembled domains and sub-domains as synthesised in 17 community capacity building studies

| **Domain** | **Sub-domains** | **Definitions** |
| --- | --- | --- |
| **Learning opportunities and skills development** |  | Skill development in relation to community capacity development involves identification of knowledge gaps [14, 15] as well as provision of opportunities for skill development [8, 14]. It includes the provision of adequate information about learning opportunities [14], access to funding [14] and networking opportunities [8] [8]. Skills development is about creating conditions inside and outside of the community that allow skills to develop and find full expression [11]. Lennie [13] refers to four types of empowerment as being integral to supporting community capacity development in relation to skills development: i) social empowerment (including new knowledge, awareness and understanding of issues, abilities and competence supporting friendships and networking); ii) political empowerment (having a voice and being listened to); iii) technological empowerment (confidence to use and speak about, information and communication technology); and, iv) psychological empowerment (self-confidence, self-esteem, feeling more motivated and respected, well-being, happiness and freedom to do things or express yourself) [13]. |
| **Resource mobilization** |  | Resource is primarily used to refer to funding [12, 17], but it also refers to availability of people, buildings, facilities and time [14]. Resource mobilization refers to the community’s ability to identify and to access external and internal resources to help achieve its vision, using the resources in new, creative and effective ways [14, 15]. It also refers to community’s ability to make decisions about fair distribution and to resolve conflict regarding distribution [14] including the distribution of common resources e.g. sharing of food [16]. |
| **Partnership/linkages/networking** |  | Partnership, also referred to as linkages with others [18] or networking or inter-sectoral participation [3, 9], refers to a group of organizations and individuals who share interests, information and resources [15] and who are working toward one or more common goals beyond the reach of any one organization or individual. Partnership is over time a dynamic and complex process where involvement varies between program area, actors and phases [3, 9] and should allow people with different levels of commitment and skills to work together and acquire confidence in their abilities as well as broaden their capacities [7] and strengthen their relationship [9]. |
| **Leadership** |  | Many leadership attributes were described including: the ability to mobilize community participation in activities [16], understand the “big picture”, articulate clear vision and facilitate consensus building and collaboration that fosters trust, respect, openness, conflict resolution, creativity, and responsibility among members [12]; acknowledging community and individual achievements; fostering the development and emergence of new leaders [14], and cultivating community input and action [17]. It also includes ability of critical reflection on process to identify areas in which the community group needs to make changes in the way they work together, and ability to foster ownership over team decisions suggesting new ideas, expressing opinions and pointing out ways to overcome obstacles [9]. |
| **Participatory decision-making** |  | Participatory or participating-decision making is defined as a way of addressing root causes of the issues identified by the community by the community being actively involved in identifying concerns and in problem solving [15]. Ability to express concerns is also a way of participating without being directly involved [16]. Ability to negotiate and resolve conflict when present is also a part of this domain [1]. |
| **Assets-based approach** |  | Defined as the unique knowledge, skills, gifts and talents possessed by community members [14]. It is also the strengths present in all individual community members as well as at the community level that forms the basis upon which the community in all of its diversity can develop its visions and act to achieve them [11]. It involves identifying skills already in the community and those needed to take action that will help achieve a vision [14]through bringing people with different knowledge and skills together [14]. |
| **Sense of community** |  | Defined as sense of place and where people do things together [14], to identify issues and solve problems and handle conflicts as a group, having trust between group members and feeling part of the community [9, 11]. It also means that community members have positive perceptions of their communities, value their diversity, celebrate together and have a sense of control and ownership in relation to planning and implementing local programmes and activities [11]. It also means respecting and valuing the viewpoints of the community members [7], understanding community history [6]and showing a sense of compassion [12]. It also refers to the extent to which participants have a shared identity in relation to the community as a physical and social environment and have a willingness to take action based on that identity [6]. |
|  | **Commitment to action** | This sub-domain of sense of community means commitment to achieving outcomes and positive change [7] and commitment to and responsibility for, improving the community [14]. |
| **Communication** |  | Communication refers to the honest and open sharing of thoughts, ideas, and information between people where everyone is informed, take responsibility to share and seek information, and has a chance to say without retaliation and censure [14]. |
|  | **Dissemination** | This sub-domain of communication means providing a continuous and appropriate feedback to the community [7] including information about best practices and lessons learnt from program- implementation. It can be evidenced through local dissemination of information through formal and informal networks, inter-professional working, linkages between practitioners and researchers, conference presentations and peer-reviewed publications. |
| **Development pathway** |  | Also named as organizational procedures [10], work procedure [7], programme management [4] and community structure and is the process of improving them and creating new ones that help community members to achieve work plans and goals [15]. The organisational structure includes standards, guidelines [8] and how tasks and responsibilities are shared [3]. |
|  | **Shared vision and clear goals** | People come together around common issues and work toward a common purpose [11]. Shared vision has been defined as “a picture of the community at some time in the future, created through true dialogue and consensus with people from all walks of life in the community, painted in enough detail that people can imagine it and believe it is possible to reach, built upon individuals’ needs, experiences, and aspirations, and inspires and motivates community members to actively reach it” [14]. |
|  | **Community needs assessment** | It refers to assessing awareness of the issues considered important among community members [15], involving community in the process of asking why [15], and including the interests and ideas of all members in the project plan [7] and project implementation and evaluation. |
|  | **Process and outcome monitoring** | It means regularly seeking feedback from participants on what worked well, what did not work well, and how project activities could be improved [13]. It includes critical reflection and analysis of successes and failures [6]. It aims to explain whether each proposed activity was carried as planned, it identifies the outcomes and states whether the process was conducted in a collaborative way [7]. |
|  | **Sustainability** | It means capability to maintain delivery of project activities through the community in addition to or instead of project partners that may have been involved in initiating the project [1]. It refers to the ability of community people to sustain a program’s focus of activity and to gain funding and resource commitment [1]. |

## Table 4 - Domains used by Group 1 authors and their correspondence with reassembled domains assessing community capacity building

| **Reference** | **Domains** | **LOSD** | **RM** | **PLN** | **L** | **PDM** | **ABA** | **SC** | **CA** | **C** | **D** | **DP** | **CNA** | **SVCG** | **S** | **POM** |
| --- | --- | --- | --- | --- | --- | --- | --- | --- | --- | --- | --- | --- | --- | --- | --- | --- |
| O’Meara | Vision and leadership |  |  |  | x |  |  |  |  |  |  |  |  | x |  |  |
|  | Structure and partnerships |  |  | x |  |  |  |  |  |  |  | x |  |  |  |  |
|  | Community engagement |  |  | x |  |  |  |  |  |  |  |  |  |  |  |  |
|  | Resources |  | x |  |  |  |  |  |  |  |  |  |  |  |  |  |
| Yeatman | Leadership |  |  |  | x |  |  |  |  |  |  |  |  |  |  |  |
|  | Partnership |  |  | x |  |  |  |  |  |  |  |  |  |  |  |  |
|  | Building, demonstrating and maintaining commitment |  |  |  |  |  |  |  | x |  |  |  |  |  | x |  |
| Andersson | Resources: mobilization and contributors |  | x |  |  |  |  |  |  |  |  |  |  |  |  |  |
|  | Leadership |  |  |  | x |  |  |  |  |  |  |  |  |  |  |  |
|  | Network: partnership, mobilization of partners and stage of development |  |  | x |  |  |  |  |  |  |  |  |  |  |  |  |
|  | Implementation: responsibility and steering committee’s role |  |  |  |  |  |  |  |  |  |  | x |  |  |  |  |
|  | Planning of activities, identification and correspondence of needs |  |  |  |  |  |  |  |  |  |  | x | x |  |  |  |
| Chilaka | Needs assessment |  |  |  |  |  |  |  |  |  |  |  | x |  |  |  |
|  | Community organization |  |  |  |  | x |  |  |  |  |  |  |  |  |  |  |
|  | Resource mobilization |  | x |  |  |  |  |  |  |  |  |  |  |  |  |  |
|  | Programme management |  |  |  |  |  |  |  |  |  |  | x |  |  |  |  |
|  | Political commitment |  |  |  |  |  |  |  | x |  |  |  |  |  |  |  |
| Ui | Community representation |  |  |  |  | x |  |  |  |  |  |  |  |  |  |  |
|  | Commitment and resource mobilization |  | x |  |  |  |  |  | x |  |  |  |  |  |  |  |
|  | Decision-making: expressing opinions, problem solving power and needs assessment |  |  |  |  | x |  |  |  |  |  | x |  |  |  |  |
|  | Management, supervision, work information shared |  |  |  |  |  |  |  |  |  |  | x |  |  |  |  |
|  | Community accountability, recognition of roles and information sharing |  |  |  |  |  |  |  |  |  | x |  |  |  |  | x |
| Parker | Resources |  | x |  |  |  |  |  |  |  |  |  |  |  |  |  |
|  | Leadership |  |  |  | x |  |  |  |  |  |  |  |  |  |  |  |
|  | Participation |  |  |  |  | x |  |  |  |  |  |  |  |  |  |  |
|  | Skills | x |  |  |  |  |  |  |  |  |  |  |  |  |  |  |
|  | Social and organizational network |  |  | x |  |  |  |  |  |  |  |  |  |  |  |  |
|  | Sense of community |  |  |  |  |  |  | x |  |  |  |  |  |  |  |  |
|  | Understanding of community history |  |  |  |  |  |  | x |  |  |  |  |  |  |  |  |
|  | Community power |  |  |  |  | x |  |  |  |  |  |  |  |  |  |  |
|  | Community reflection |  |  |  |  |  |  |  |  |  |  |  |  |  |  | x |
| Garcia | Teamwork skills | x |  |  |  |  |  |  |  |  |  |  |  |  |  |  |
| Ramirez | Understanding of the issues | x |  |  |  |  |  |  |  |  |  |  |  |  |  |  |
|  | Productive team work |  |  | x |  |  |  |  |  |  |  |  |  |  |  |  |
|  | Goals achieved due to team work collaboration |  |  | x |  |  |  |  |  |  |  |  |  |  |  |  |
|  | Members’ viewpoints respected and valued |  |  |  |  |  |  | x |  |  |  |  |  |  |  |  |
|  | Capability of influencing workgroup | x |  |  |  |  |  |  |  |  |  |  |  |  |  |  |
|  | Participation of all members is encouraged |  |  |  |  | x |  |  |  |  |  |  |  |  |  |  |
|  | Identification and strengthen of skills |  |  |  |  |  | x |  |  |  |  |  |  |  |  |  |
|  | Learning to overcome difficulties | x |  |  |  |  |  |  |  |  |  |  |  |  |  |  |
|  | Positive and open atmosphere |  |  |  |  |  |  |  |  |  |  | x |  |  |  |  |
|  | Effective conflict-management atmosphere |  |  |  |  |  |  |  |  |  |  | x |  |  |  |  |
|  | Shared goals |  |  |  |  |  |  |  |  |  |  |  |  | x |  |  |
|  | Participation in decision-making process |  |  |  |  | x |  |  |  |  |  |  |  |  |  |  |
|  | Interests and ideas from all |  |  |  |  |  |  |  |  |  |  |  | x |  |  |  |
|  | Individual differences taken into account |  |  |  |  |  |  |  |  |  |  |  | x |  |  |  |
|  | Relationships with communities |  |  | x |  |  |  |  |  |  |  |  |  |  |  |  |
|  | Social networking |  |  | x |  |  |  |  |  |  |  |  |  |  |  |  |
|  | Democratic leadership |  |  |  | x |  |  |  |  |  |  |  |  |  |  |  |
|  | Leader with adequate conflict-resolution skills |  |  |  | x |  |  |  |  |  |  |  |  |  |  |  |
|  | Work procedures |  |  |  |  |  |  |  |  |  |  | x |  |  |  |  |
|  | Communication |  |  |  |  |  |  |  |  | x |  |  |  |  |  |  |
|  | Shared information and solved problems by consensus |  |  |  |  | x |  |  |  |  | x |  |  |  |  |  |
|  | Optimized use of resources |  | x |  |  |  |  |  |  |  |  |  |  |  |  |  |
|  | Continuous improvement and feedback |  |  |  |  |  |  |  |  |  |  |  |  |  |  | x |
|  | Deadlines respected |  |  |  |  |  |  |  | x |  |  |  |  |  |  |  |
| Robins | Growing the knowledge base | x |  |  |  |  |  |  |  |  |  |  |  |  |  |  |
|  | Access infrastructure and resources |  | x |  |  |  |  |  |  |  |  |  |  |  |  |  |
|  | Develop and sustain networks and relationships |  |  | x |  |  |  |  |  |  |  |  |  |  | x |  |
|  | Build the information base and tools |  |  |  |  |  |  |  |  |  | x |  |  |  |  |  |
|  | Strengthen institutional structures and process |  |  |  |  |  |  |  |  |  |  |  |  |  |  | x |
|  | Underpin with effective business system |  |  |  |  |  |  |  |  |  |  | x |  |  |  |  |
|  | Definition of organizational business |  |  |  |  |  |  |  |  |  |  |  |  | x |  |  |
|  | Set of standard and expectations |  |  |  |  |  |  |  |  |  |  |  |  | x |  |  |
| Schulz | Group empowerment |  |  |  |  | x |  |  |  |  |  |  |  |  |  |  |
|  | Personal organization and community benefits of participation |  |  |  |  | x |  |  |  |  |  |  |  |  |  |  |
|  | Member background and meeting attendance |  |  |  |  | x |  |  |  |  |  |  |  |  |  |  |
|  | Community empowerment | x |  |  |  |  |  |  |  |  |  |  |  |  |  |  |
|  | Decision-making procedures |  |  |  |  | x |  |  |  |  |  |  |  |  |  |  |
|  | Working together as a group to solve problems |  |  |  |  |  |  |  | x |  |  |  |  |  |  |  |
|  | Level of influence and power of self and others in the group |  |  |  |  | x |  |  |  |  |  |  |  |  |  |  |
|  | Perceived level of trust |  |  |  |  |  |  | x |  |  |  |  |  |  |  |  |
|  | General satisfaction |  |  |  |  |  |  | x |  |  |  |  |  |  |  |  |
|  | Sense of ownership belonging to the group |  |  |  |  |  |  | x |  |  |  |  |  |  |  |  |
|  | Comfort level for expressing opinions |  |  |  |  |  |  |  |  | x |  |  |  |  |  |  |
|  | Accomplishments / impacts of the group |  |  |  |  |  |  |  |  |  |  |  |  |  | x |  |
|  | Meeting organization, agenda setting, facilitation and staffing |  |  |  |  |  |  |  |  |  |  | x |  |  |  |  |
|  | Addressing conflict problems |  |  |  |  | x |  |  |  |  |  |  |  |  |  |  |
|  | Leadership and participation |  |  |  | x |  |  |  |  |  |  |  |  |  |  |  |
| South | Community diversity |  |  |  |  |  | x |  |  |  |  |  |  |  |  |  |
|  | Organizational procedures |  |  |  |  |  |  |  |  |  |  | x |  |  |  |  |
|  | Communication and flow of information |  |  |  |  |  |  |  |  | x | x |  |  |  |  |  |
|  | Staff support to engage with communities |  |  | x |  |  |  |  |  |  |  |  |  |  |  |  |
|  | Opportunities in decision making process |  |  |  |  | x |  |  |  |  |  |  |  |  |  |  |
|  | Resources access and control by communities |  | x |  |  |  |  |  |  |  |  |  |  |  |  |  |

LOSD = Learning opportunities and skills development; RM = Resource mobilization; PLN = Partnership/linkages/networking/; L = Leadership; PDM = Participatory decision-making; ABA = Assets-based approach; SC = Sense of community; CA = Commitment to action; C = Communication; D = Dissemination; DP = Development pathway; CNA = Community needs assessment; SVCG = Shared vision and clear goals; S = Sustainability; POM = Process and outcome monitoring

**Table 5 - Domains used by Group 2 authors and their correspondence with the reassembled domains assessing** community capacity building

| **Reference** | **Domains** | **LOSD** | **RM** | **PLN** | **L** | **PDM** | **ABA** | **SC** | **CA** | **C** | **D** | **DP** | **CNA** | **SVCG** | **S** | **POM** |
| --- | --- | --- | --- | --- | --- | --- | --- | --- | --- | --- | --- | --- | --- | --- | --- | --- |
| Jackson | Community is supportive of diversity |  |  |  |  |  | x |  |  |  |  |  |  |  |  |  |
|  | Positive perceptions of their communities |  |  |  |  |  |  | x |  |  |  |  |  |  |  |  |
|  | Residents celebrate together |  |  |  |  |  |  | x |  |  |  |  |  |  |  |  |
|  | Active participation in the social, political and economic life of the community |  |  |  |  | x |  |  |  |  |  |  |  |  |  |  |
|  | Work together towards a common purpose |  |  | x |  |  |  |  |  |  |  |  |  | x |  |  |
|  | Participation from all parts of the community |  |  |  |  | x |  |  |  |  |  |  |  |  |  |  |
|  | Sense of control and sense of ownership of programs |  |  |  |  |  |  | x |  |  |  |  |  |  |  |  |
| Lempa | Leadership |  |  |  | x |  |  |  |  |  |  |  |  |  |  |  |
|  | Resources |  | x |  |  |  |  |  |  |  |  |  |  |  |  |  |
|  | External networking |  |  | x |  |  |  |  |  |  |  |  |  |  |  |  |
|  | Visibility / recognition |  |  |  |  |  |  |  |  |  |  |  |  |  | x |  |
|  | ability and commitment to organizing action |  |  |  |  |  |  |  | x |  |  |  |  |  |  |  |
|  | Personnel sustainability |  |  |  |  |  |  |  |  |  |  |  |  |  | x |  |
|  | Communication with community members |  |  |  |  |  |  |  |  | x |  |  |  |  |  |  |
|  | Relationship with influential others |  |  | x |  |  |  |  |  |  |  |  |  |  |  |  |
| Lennie | Social empowerment |  |  |  |  |  |  |  |  |  |  |  |  |  |  |  |
|  | Political empowerment |  |  |  |  |  |  |  |  |  |  |  |  |  |  |  |
|  | Technological empowerment |  |  |  |  |  |  |  |  |  |  |  |  |  |  |  |
|  | Psychological empowerment |  |  |  |  |  |  |  |  |  |  |  |  |  |  |  |
| Littlejohns | Shared vision |  |  |  |  |  |  |  |  |  |  |  |  | x |  |  |
|  | Living together in a healthy and sustainable way |  |  | x |  |  |  |  |  |  |  |  |  |  |  |  |
|  | Resources |  | x |  |  |  |  |  |  |  |  |  |  |  |  |  |
|  | Knowledge and skills | x |  |  |  |  |  |  |  |  |  |  |  |  |  |  |
|  | Participation |  |  |  |  | x |  |  |  |  |  |  |  |  |  |  |
|  | Leadership |  |  |  | x |  |  |  |  |  |  |  |  |  |  |  |
|  | Communication |  |  |  |  |  |  |  |  | x |  |  |  |  |  |  |
|  | Critical learning |  |  |  |  |  |  |  |  |  |  |  |  |  |  | x |
| Maclellan-Wright | Participation |  |  |  |  | x |  |  |  |  |  |  |  |  |  |  |
|  | Leadership |  |  |  | x |  |  |  |  |  |  |  |  |  |  |  |
|  | Community structures |  |  |  |  |  |  |  |  |  |  | x |  |  |  |  |
|  | Role of external supports |  |  | x |  |  |  |  |  |  |  |  |  |  |  |  |
|  | Asking why |  |  |  |  | x |  |  |  |  |  |  |  |  |  |  |
|  | Resource mobilization |  | x |  |  |  |  |  |  |  |  |  |  |  |  |  |
|  | Skills, knowledge and learning | x |  |  |  |  |  |  |  |  |  |  |  |  |  |  |
|  | Links with others |  |  | x |  |  |  |  |  |  |  |  |  |  |  |  |
|  | Sense of community |  |  |  |  |  |  | x |  |  |  |  |  |  |  |  |
| Yassi | Opportunity to participate |  |  |  |  | x |  |  |  |  |  |  |  |  |  |  |
|  | Linkage systems |  |  | x |  |  |  |  |  |  |  |  |  |  |  |  |
|  | Information and communication patterns |  |  |  |  |  |  |  |  | x |  |  |  |  |  |  |
|  | Joint decision making |  |  |  |  | x |  |  |  |  |  |  |  |  |  |  |
|  | mobilization of people and resources |  | x |  |  |  |  |  |  |  |  |  |  |  |  |  |
|  | self-definition of interests by the local community |  |  |  |  |  |  |  |  |  |  |  | x |  |  |  |
| Zacocks | Resource (funding) |  | x |  |  |  |  |  |  |  |  |  |  |  |  |  |
|  | lead agency (supportive, power broker) |  |  | x |  |  |  |  |  |  |  |  |  |  |  |  |
|  | Governance (stability, participation from organizational representatives, participatory decision making) |  |  |  |  | x |  |  |  |  |  |  |  |  |  |  |
|  | Leadership (collaborative style, project director effectiveness, stability) |  |  |  | x |  |  |  |  |  |  |  |  |  |  |  |

LOSD = Learning opportunities and skills development; RM = Resource mobilization; PLN = Partnership/linkages/networking/; L = Leadership; PDM = Participatory decision-making; ABA = Assets-based approach; SC = Sense of community; CA = Commitment to action; C = Communication; D = Dissemination; DP = Development pathway; CNA = Community needs assessment; SVCG = Shared vision and clear goals; S = Sustainability; POM = Process and outcome monitoring

(3):202-212.

(3):202-212.
